# Ethyl Gallate Induces Apoptosis of HL-60 Cells by Promoting the Expression of Caspases-8, -9, -3, Apoptosis-Inducing Factor and Endonuclease G

**DOI:** 10.3390/ijms130911912

**Published:** 2012-09-20

**Authors:** Woong-Hyun Kim, Hyun-Ok Song, Hwa-Jung Choi, Ho-Il Bang, Du-Young Choi, Hyun Park

**Affiliations:** 1 Department of Infection Biology, Zoonosis Research Center, Wonkwang University School of Medicine, 344-2, Shinyong-dong, Iksan, Chonbuk 570-749, South Korea; E-Mails: woong621@gmail.com (W.-H.K.); sea5328@daum.net (H.-O.S.); rerived@empal.com (H.-J.C.); 2 Department of Pediatrics, Wonkwang University School of Medicine, 344-2, Shinyong-dong, Iksan, Chonbuk 570-749, South Korea; E-Mail: bang@medigate.net

**Keywords:** ethyl gallate, apoptosis, death receptor, mitochondrial-mediated pathways

## Abstract

Many phytochemicals have been recognized to have potential therapeutic efficacy in cancer treatment. In this study, we investigated ethyl gallate (EG) for possible proapoptotic effects in the human promyelocytic leukemia cell line, HL-60. We examined cell viability, morphological changes, DNA content and fragmentation, and expression of apoptosis-related proteins for up to 48 h after EG treatment. The results showed that EG induced morphological changes and DNA fragmentation and reduced HL-60 cell viability in a dose-dependent and time-dependent manner. Western blotting analysis indicated that EG-mediated HL-60 apoptosis mainly occurred through the mitochondrial pathway, as shown by the release of cytochrome *c*, apoptosis-inducing factor (AIF), and endonuclease G (Endo G), as well as the upregulation of Bcl-2-associated X protein (Bax). EG also activated the death receptor-dependent pathway of apoptosis by enhancing the expression of caspases-8, -9, and -3 and the Bcl-2 interacting domain (Bid). Collectively, our results showed that EG induces apoptosis in HL-60 via mitochondrial-mediated pathways.

## 1. Introduction

Many anticancer agents have been reported to mediate their activity through mitochondrial events such as depletion of glutathione, production of reactive oxygen species, and activation of intrinsic apoptosis pathways (for example, cytochrome *c*, Apaf-1, and caspase-9) [1–3]. Over the years, natural products have played a significant role in the development of anticancer drugs [4,5].

Apoptosis is a central mechanism to eliminate unwanted cells that may accumulate during physiological processes and pathologic conditions such as cancer and autoimmune disease [6]. During apoptosis, intrinsic death signals activate caspase-9 via a mitochondrial-dependent complex [7]. Caspase-3 is activated by caspase-8 and caspase-9, and it participates in apoptosis by cleaving cellular proteins [8].

Mitochondria are involved in a variety of cellular processes and functions, such as cell differentiation, growth, survival, and apoptosis [9,10]. Mitochondria are key organelles in the development of anticancer therapeutics that promote cell death [11–13]. Most approaches focus on 2 mitochondrial-based strategies. One is to target the mitochondria directly, for example, with agents that act on the Bcl-2 family members. The second approach is to induce cancer cell apoptosis indirectly through mitochondrial-mediated pathways [14].

Paclitaxel (Taxol^®^) is a naturally occurring microtubule-stabilizing anticancer drug isolated from the bark of the Pacific yew tree (*Taxus brevifolia*). Although paclitaxel is currently considered one of the most important chemotherapeutic agents [15], it can cause severe side effects such as allergic shock, hypotension, neurotoxicity, and nephrotoxicity [16,17].

*Galla Rhois* extract is a mixture of polygalloyl esters of glucose that contains gallotannins such as ethyl gallate (EG), methyl gallate, and gallic acid, and it has been reported to have various biological activities, including antibacterial [18], antimetastatic or anti-invasive effects [19], as well as anti-apoptotic and anti-necrotic protective effects on liver cells [20]. EG is used as a food additive and has been reported to exhibit anticancer [21], antimicrobial [9], and free radical scavenging activities [22]. In this study, we show that EG isolated from *Galla Rhois* has anticancer activity against a human leukemia cell line. Mechanistic studies show that EG acts on mitochondrial-dependent pathways and the caspase cascade to activate the intrinsic apoptotic pathway through expression of caspases-8, -9, and -3; apoptosis-inducing factor (AIF); and endonuclease G (Endo G).

## 2. Results and Discussion

### 2.1. Effect of EG on the Morphology and Viability of HL-60 Cells

**Figure 1:**
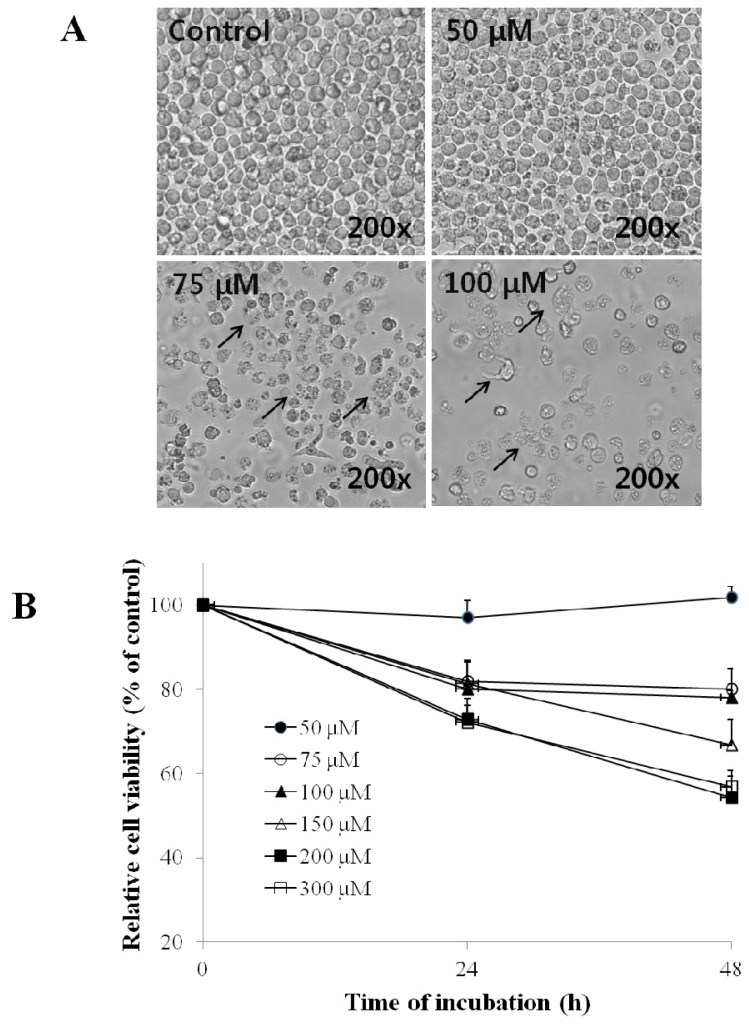
Effect of ethyl gallate (EG) on the morphology and viability of HL-60 cells. Cells were cultured with 50 μM, 75 μM, 100 μM, 150 μM, 200 μM, or 300 μM EG for 24 h or 48 h. (**A**) The morphological changes of HL-60 cells were visualized by phase-contrast microscopy (200×); (**B**) Cell viability was measured by the methanethiosulfonate/phenazine methosulfate solution (MTS) assay. Each point is the mean ± SD of 3 experiments.

After treatment for 24 h or 48 h with EG, HL-60 cells showed changes in morphology, including shrinkage of the cell membrane and the development of apoptotic bodies (Figure 1A). Consistent with these effects, the viability of EG-treated cells decreased in a time- and dose-dependent manner (Figure 1B), demonstrating that EG has a cytotoxic effect on HL-60 cells.

### 2.2. Analysis of EG-Induced Apoptosis in HL-60 Cells

**Figure 2:**
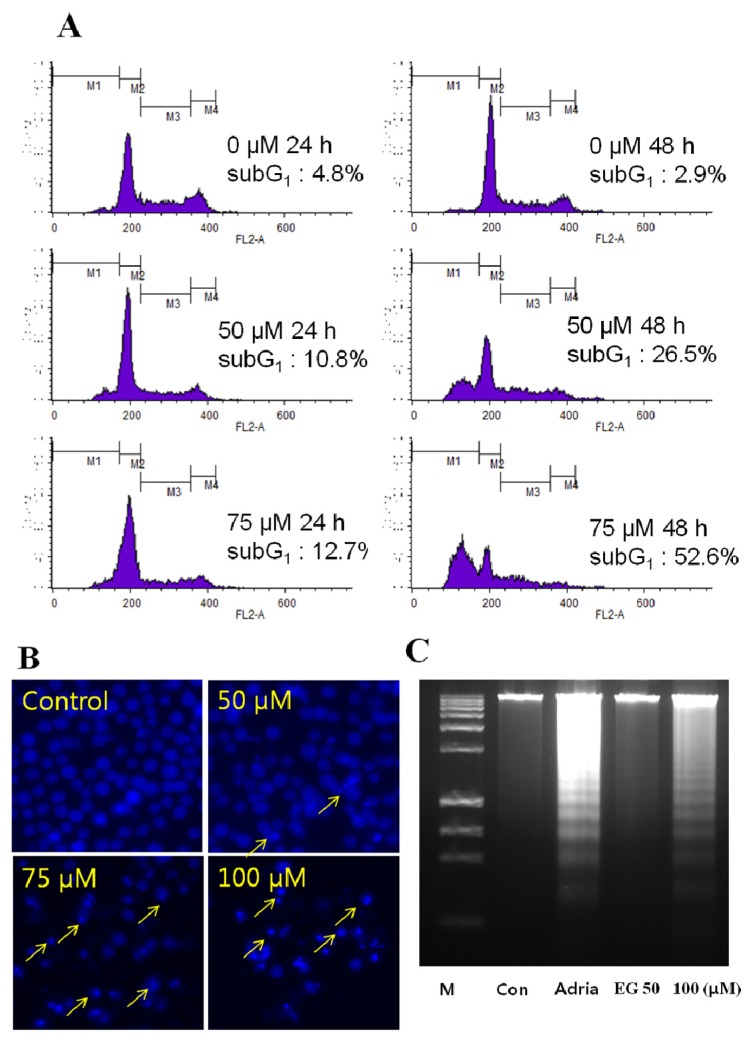
Effect of EG on the cell cycle and induction of apoptosis in HL-60 cells. Cells were incubated with 50 μM or 75 μM EG for 24 h or 48 h. (**A**) Cells were examined for DNA content by staining with Propidium iodide (PI) and analysis by flow cytometry. M1: Sub G1 phase, M2: G1 phase, M3: S phase, M4: G2/M phase; (**B**) Cells were fixed, stained with DAPI, and examined by fluorescence microscopy; (**C**) DNA fragmentation was examined by 1.0% agarose gel electrophoresis of genomic DNA, followed by ethidium bromide staining. M: DNA molecular weight marker, Con: control, Adria: adriamycin. These results presented are representative of data obtained from three independent experiments carried out in triplicate.

To determine whether the cytotoxicity of EG for HL-60 cells was a result of apoptosis, we analyzed the DNA content and cell cycle distribution of EG-treated cells by flow cytometric analysis of PI-stained cells. EG treatment increased the proportion of cells in subG1 phase, indicative of a reduction in DNA content, in a concentration- and time-dependent manner. Treatment of cells for 24 h or 48 h with 50 μM or 75 μM EG increased the percentage of cells in the subG1 phase from a baseline of 2.9% to 26.5% or 52.6%, respectively (Figure 2A). Similarly, HL-60 cells treated with 50 μM, 75 μM, or 100 μM EG for 48 h showed a dose-dependent increase in apoptosis, as measured by the DAPI assay (Figure 2B). Finally, DNA fragmentation in the EG-treated cells was confirmed by agarose gel electrophoresis, which showed the presence of DNA laddering, a marker of apoptosis, in EG-treated HL-60 cells (Figure 2C).

### 2.3. Effect of EG on the Expression of Apoptosis-Associated Proteins in HL-60 Cells

**Figure 3:**
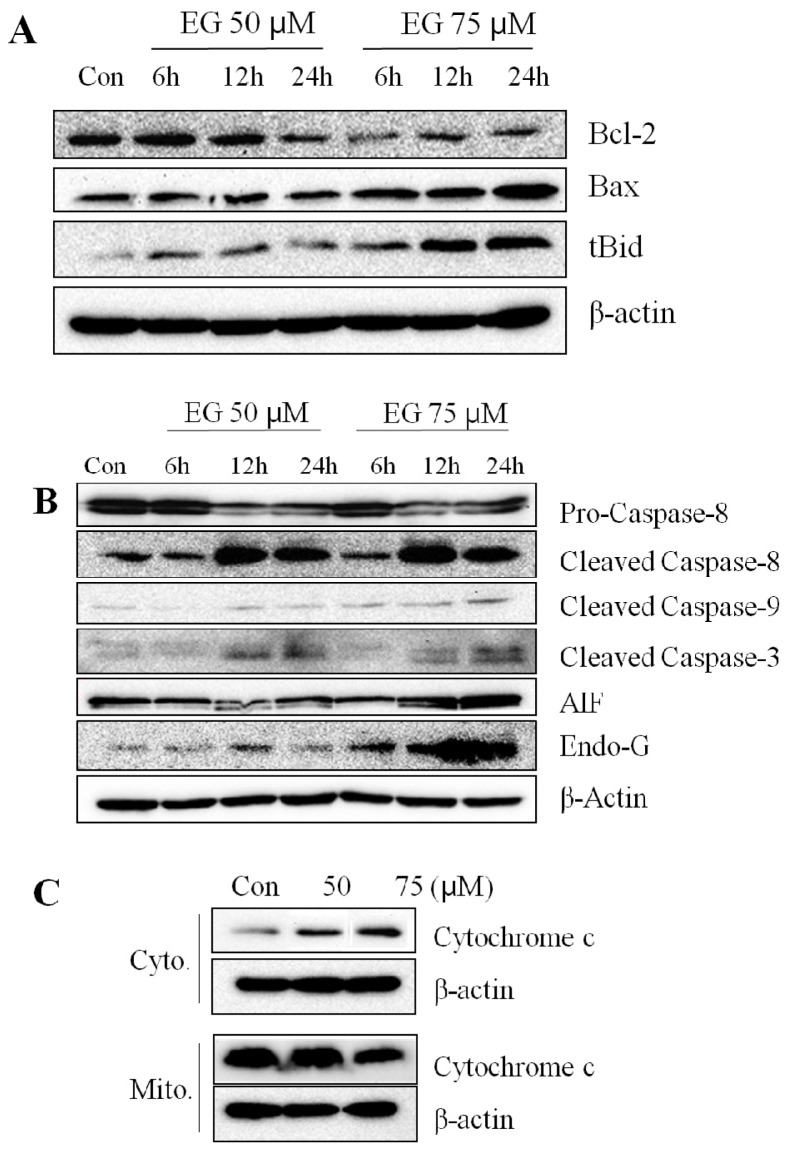
Effect of EG on the expression of apoptosis-associated proteins in HL-60 cells. HL-60 cells (5 × 10^5^ cells) were treated with 50 μM or 75 μM EG for 6 h, 12 h, or 24 h. Cell lysates were resolved by SDS-PAGE and subjected to western blotting. (**A**) Bcl-2, Bax, and tBid. Con: Control, Cyto: cytosol, Mito: mitochondria; (**B**) Caspase-8, caspase-9, caspase-3, apoptosis-inducing factor (AIF), and Endo G; (**C**) Cytochrome *c*. These results presented are representative of data obtained from three independent experiments carried out in triplicate. These results presented are representative of data obtained from three independent experiments carried out in triplicate.

To investigate the molecular mechanism by which EG induced apoptosis in HL-60 cells, we examined the expression of several apoptosis-associated proteins by western blotting. We found that EG treatment of HL-60 cells decreased the expression of Bcl-2 at 75 μM EG, and increased Bax and truncated Bid (tBid) expression at 24 h (Figure 3A). Also, caspase-9 expression increased after treatment of 75 μM EG for 24 h (Figure 3B). Caspase-8 expression did not increase until 6 h after treatment of 50 μM or 75 μM EG and increased at 12 h after treatment of 50 μM or 75 μM EG (Figure 3B). Caspase-3 expression increased at 12 h and 24 h after treatment of 50 μM or 75 μM EG (Figure 3B). AIF, Endo G and cytochrome *c* expression increased at 24 h after treatment of 75 μM EG (Figure 3B,C). These results suggest that EG induced apoptotic death in HL-60 cells via activation of the caspase enzyme cascade and through mitochondrial pathways.

Gallotannin and other tannins have been reported to have a variety of biological effects, including anti-inflammatory, anticancer, and antiviral effects [23–25]. One study showed that galloylglucose inhibits gelatinase-mediated degradation of type IV collagens, thereby inhibiting metastatic tumor cell invasion through the extracellular matrix [26]. In another report, gallic acid induced G0/G1 cell cycle arrest and apoptosis in HL-60 cells by inhibiting cyclin D and E and by activating mitochondrial-dependent pathways [27].

The present study showed that EG decreased the viability of HL-60 cells (Figure 1B) by the induction of apoptosis (Figure 1A). EG dose-dependently induced DNA fragmentation and apoptosis, as revealed by increases in the subG1 DNA content of the cells (Figure 2A), DAPI staining (Figure 2B), and increased DNA laddering on agarose gels (Figure 2C).

The role of Bcl-2 family proteins in EG-induced apoptosis was investigated by examining their expression. Bax and tBid levels were shown to increase, while Bcl-2 expression decreased (Figure 3A). Our findings are consistent with the known pattern of events occurring in apoptosis, in which an increase in the ratio of Bax to Bcl-2 stimulates the release of cytochrome *c* from the mitochondria into the cytosol. Cytochrome *c* promotes caspase-9 activation and binding to apoptotic protease activating factor-1 (APAF-1), which leads to the activation of caspase-3.

Caspases play a key role in the initiation and execution of apoptosis [28]. In this study, caspases-3, -8, and -9 were activated in EG-treated HL-60 cells. Activation of caspase-8 by death receptors results in the cleavage and activation of the effector caspase-3. Caspase-8 also cleaves Bid, which induces cytochrome *c* efflux from the mitochondria and subsequent activation of caspases-9 and -3, as described above [29]. Our results indicate that EG induced the cleavage of full-length Bid to generate tBid, which translocates to the mitochondria.

Mitochondria play a crucial role in many apoptotic responses through the caspase-independent release of apoptogenic proteins such as AIF and EndoG [30]. AIF and EndoG are released into the cytosol and translocate to the nucleus where they induce chromatin condensation and extensive DNA fragmentation [31]. Previous reports have shown that Bcl-2 overexpression prevents AIF release and consequently reduces cell death [31,32]. This is consistent with the present study, which shows that a decrease in the Bcl-2 protein level by EG treatment may promote the release of AIF from the mitochondria. Although further studies are needed, the present work suggests that mitochondrial dysfunction may be a good surrogate biomarker for assessing the antitumor activity of EG in human leukemia cells.

## 3. Materials and Methods

### 3.1. Chemicals and Reagents

**Figure 4:**
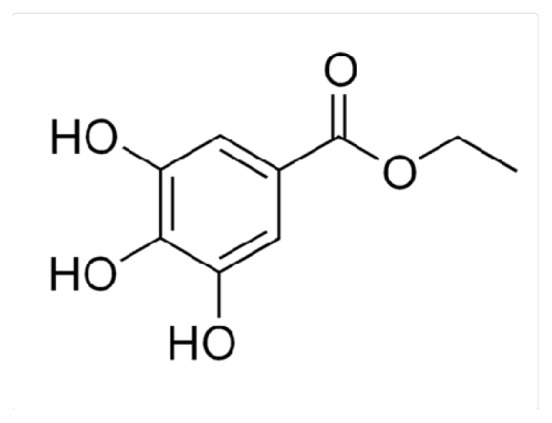
Chemical structure of ethyl gallate.

Propidium iodide (PI), dimethyl sulfoxide (DMSO), ribonuclease A (RNase A), and 4,6-diamidino-2-phenylindole dihydrochloride (DAPI) were obtained from Sigma-Aldrich Corp. (St. Louis, MO, USA). Antibodies specific for the following proteins were purchased from the indicated sources. Caspase-3 (Cell Signaling Technology; Danvers, MA, USA); caspase-8, caspase-9, and Endo G (Enzo Life Sciences; Farmingdale, NY, USA); AIF (Bethyl Laboratories; Montgomery, TX, USA); B-cell lymphoma 2 (Bcl-2), Bcl-2-associated X protein (Bax), and Bcl-2-interacting domain (Bid) (Santa Cruz Biotechnology; Santa Cruz, CA, USA); cytochrome *c* (BioVision; Conesa, Buenos Aires, Argentina); and β-actin (Sigma-Aldrich; St. Louis, MO, USA). RPMI 1640 medium, penicillin-streptomycin, fetal bovine serum (FBS), and l-glutamine were obtained from Gibco-BRL (Grand Island, NY, USA). Ethyl gallate isolated from *Galla Rhois* was obtained from Dr. Youn-Chul Kim’s Laboratory at the College of Pharmacy, Wonkwang University, Iksan, South Korea (Figure 4). All chemicals were of reagent grade.

### 3.2. Cell Culture

The human promyelocytic leukemia cell line HL-60 was obtained from the American Type Culture Collection (ATCC; Manassas, VA, USA). Cells were grown in 75 cm^2^ tissue culture flasks in RPMI 1640 medium supplemented with 10% FBS, 100 U/mL penicillin, 100 μg/mL streptomycin, and 2 mM l-glutamine and maintained at 37 °C in a humidified 5% CO_2_ atmosphere.

### 3.3. Cell Viability

HL-60 cells were cultured to 80% confluence in 24-well tissue culture plates. 1 mL of 100 mM EG diluted with 1 mL RPMI 1640 medium to adjust to pH 7.2 values. Varying concentrations (0 μM, 50 μM, 75 μM, 100 μM, 150 μM, 200 μM, and 300 μM) of EG were added to the wells and the cells were incubated for 24 h or 48 h at 37 °C. Cell morphology was then observed using a phase-contrast microscope at 200× magnification (Olympus; Hamburg, Germany) and images were recorded. Cell viability assays were performed using the CellTiter 96^®^ AQueous One Solution Cell Proliferation Assay (Promega; Madison, WI, USA) according to the manufacturer’s instructions. An aliquot of the methanethiosulfonate/phenazine methosulfate solution (MTS) was added to each well, and the cells were incubated at 37 °C for 1.5 h. The absorbance was read at 490 nm wavelength using a Tecan Infinite F200 microplate reader (Tecan; Männedorf, Switzerland).

### 3.4. Cell Cycle Analysis by Flow Cytometry and DAPI Staining of Apoptotic Cells

HL-60 cells (1 × 10^6^) were treated with 50 μM or 75 μM EG for 24 h or 48 h at 37 °C. Cells were then harvested by centrifugation and fixed in 70% ethanol at 4 °C for 24 h. Fixed cells were resuspended in PBS containing 40 μg/mL PI, 100 μg/mL RNase A, and 0.1% Triton X-100 and incubated in the dark for 30 min at room temperature. Cell cycle distribution was analyzed by flow cytometry on a FACSCalibur (BD Biosciences; San Jose, CA, USA). To investigate apoptotic cells, HL-60 cells (1 × 10^6^) incubated with different concentration of 50 μM, 75 μM and 100 μM EG for 24 h or 48 h at 37 °C, and then DAPI staining conducted as described previously [33]. The cells were photographed using a fluorescence microscopy.

### 3.5. DNA Fragmentation Analysis

HL-60 cells were seeded at 1 × 10^6^ cells per well in 24-well plates and incubated with different concentrations of EG (0 μM, 50 μM, or 200 μM) for 24 h. The cells were washed twice in ice-cold PBS and then harvested by centrifugation. DNA was extracted from the cell pellets using DNeasy Blood & Tissue Kit (Qiagen; Hilden, Germany) according to the manufacturer’s instructions. DNA was separated by 1% agarose gel electrophoresis, and DNA fragments were visualized by ethidium bromide staining and digitized imaging of the gel.

### 3.6. Western Blotting Analysis

The expression of apoptosis-related proteins (caspases-8, -9, -3; AIF; Endo G; Bid; Bax; and Bcl-2) in HL-60 cells was determined by sodium dodecyl sulfate-polyacrylamide gel electrophoresis (SDS-PAGE) of lysates followed by western blotting. For this, HL-60 cells (1.5 × 10^6^) were treated with 50 μM or 75 μM EG for 6 h, 12 h, or 24 h. Total cell lysates were obtained by resuspending cells in ice-cold radioimmunoprecipitation assay (RIPA) buffer [50 mM Tris-HCl (pH 7.8), 150 mM NaCl, 1% Triton X-100, 0.5% sodium deoxycholate, and 0.1% sodium dodecyl sulfate] for 30 min followed by centrifugation. Protein concentration was determined using a NanoDrop spectrophotometer (NanoDrop Technologies; Silverside Rd, WA, USA). Aliquots of lysates (100 μg protein equivalents) were resolved by 12% SDS-PAGE and transferred onto nitrocellulose membranes (Bio-Rad; Gangnam ku, Seoul, Korea). The membranes were blocked and incubated with the indicated primary antibodies and then with horseradish peroxidase-conjugated secondary antibodies. Blots were visualized using an enhanced chemiluminescence (ECL) kit (Amersham; Kangnam-ku, Seoul, Korea) and imaged using the FluorChemE imaging system (Cell Biosystems; St. New Montgomery, CA, USA).

To measure release of cytochrome *c* from HL-60 mitochondria, cell lysates were separated using a mitochondrial/cytosol fractionation kit (BioVision; Conesa, Buenos Aires, Argentina). In brief, 2 × 10^7^ cells were harvested by centrifugation at 600× *g* for 5 min and washed twice with cold PBS. The pellets were resuspended in 500 μL of extraction buffer containing dithiothreitol and a protease inhibitor mixture (BioVision) and incubated on ice for 10 min. The cells were then homogenized at 4 °C using a Dounce tissue grinder and centrifuged at 700× *g* for 10 min at 4 °C. The supernatant was collected and re-centrifuged at 10,000× *g* for 30 min at 4 °C. The resulting supernatant was harvested and designated as the cytosolic fraction. The pellet was resuspended in an appropriate buffer and designated as the mitochondrial fraction. Cytochrome *c* expression in the fractions was analyzed by western blotting as indicated above.

## 4. Conclusion

The present study described that EG isolated from *Galla Rhois* has anticancer activity against a human leukemia cell line acting on mitochondrial-dependent pathways and the caspase cascade to activate the intrinsic apoptotic pathway through expression of caspases-8, -9, and -3; apoptosis-inducing factor (AIF); and endonuclease G (Endo G). It will be interesting to further investigate the anticancer activity of the EG in preventing various cancer mediated injuries in *in vivo* pathological situations.

## References

[1] Rakshit S., Mandal L., Pal B.C., Bagchi J., Biswas N., Chaudhuri J., Chowdhury A.A., Manna A., Chaudhuri U., Konar A. (2010). Involvement of ROS in chlorogenic acid-induced apoptosis of Bcr-Abl+ CML cells. Biochem. Pharmacol.

[2] Hsu J.L., Pan S.L., Ho Y.F., Hwang T.L., Kung F.L., Guh J.H. (2011). Costunolide induces apoptosis through nuclear calcium2+ overload and DNA damage response in human prostate cancer. J. Urol.

[3] Indran I.R., Tufo G., Pervaiz S., Brenner C. (2011). Recent advances in apoptosis, mitochondria and drug resistance in cancer cells. Biochim. Biophys. Acta Bioenerg.

[4] Newman D.J., Cragg G.M., Snader K.M. (2003). Natural products as sources of new drugs over the period 1981–2002. J. Nat. Prod.

[5] Gupta S.C., Kim J.H., Prasad S., Aggarwal B.B. (2010). Regulation of survival, proliferation, invasion, angiogenesis, and metastasis of tumor cells through modulation of inflammatory pathways by nutraceuticals. Cancer Metastasis Rev.

[6] Kandasamy K., Srinivasula S.M., Alnemri E.S., Thompson C.B., Korsmeyer S.J., Bryant J.L., Srivastava R.K. (2003). Involvement of proapoptotic molecules Bax and Bak in tumor necrosis factor-related apoptosis-inducing ligand (TRAIL)-induced mitochondrial disruption and apoptosis: Differential regulation of cytochrome *c* and Smac/DIABLO release. Cancer Res.

[7] Budihardjo I., Oliver H., Lutter M., Luo X., Wang X.D. (1999). Biochemical pathways of caspase activation during apoptosis. Annu. Rev. Cell Dev. Biol.

[8] Earnshaw W.C., Martins L.M., Kaufmann S.H. (1999). Mammalian caspases: Structure, activation, substrates, and functions during apoptosis. Annu. Rev. Cell Dev. Biol.

[9] Shibata H., Kondo K., Katsuyama R., Kawazoe K., Sato Y., Murakami K., Shibata H., Kondo K., Katsuyama R., Kawazoe K. (2005). Alkyl gallates, intensifiers of beta-lactam susceptibility in methicillin-resistant *Staphylococcus aureus*. Antimicrob. Agents Chemother.

[10] Lakshminarasimhan M., Steegborn C. (2011). Emerging mitochondrial signaling mechanisms in physiology, aging processes, and as drug targets. Exp. Gerontol.

[11] Gogvadze V., Orrenius S., Zhivotovsky B. (2009). Mitochondria as targets for cancer chemotherapy. Semin. Cancer Biol.

[12] Mayevsky A. (2009). Mitochondrial function and energy metabolism in cancer cells: Past overview and future perspectives. Mitochondrion.

[13] Chiang P.C., Lin S.C., Pan S.L., Kuo C.H., Tsai I.L., Kuo M.T., Wen W.C., Chen P., Guh J.H. (2010). Antroquinonol displays anticancer potential against human hepatocellular carcinoma cells: A crucial role of AMPK and mTOR pathways. Biochem. Pharmacol.

[14] Leber B., Geng F., Kale J., Andrews D.W. (2010). Drugs targeting Bcl-2 family members as an emerging strategy in cancer. Expert Rev. Mol. Med.

[15] Rowinsky E.K., Onetto N., Canetta R.M., Arbuck S.G. (1992). Taxol: The first of the taxanes, an important new class of antitumor agents. Semin. Oncol.

[16] Weiss R.B., Donehower R.C., Wiernik P.H., Ohnuma T., Gralla R.J., Trump D.L., Baker J.J.R., van Echo D.A, von Hoff D.D., Leyland-Jones B. (1990). Hypersensitivity reactions from taxol. J. Clin. Oncol..

[17] Gelderblom H., Verweij J., Nooter K., Sparreboom A., Cremophor EL (2001). The drawbacks and advantages of vehicle selection for drug formulation. Eur. J. Cancer.

[18] Ahn Y.J., Lee C.O., Kweon J.H., Ahn J.W., Park J.H. (1998). Growth-inhibitory effects of *Galla Rhois* derived tannins on intestinal bacteria. J. Appl. Microbiol.

[19] Ata N., Oku T., Hattori M., Fujii H., Nakajima M., Saiki I. (1996). Inhibition by galloylglucose (GG6-10) of tumor invasion through extracellular matrix and gelatinase-mediated degradation of type IV collagens by metastatic tumor cells. Oncol. Res.

[20] Park E.J., Zhao Y.Z., An R.B., Kim Y.C., Sohn D.H. (2008). 1,2,3,4,6-Penta-*O*-galloyl-beta-d-glucose from *Galla Rhois* protects primary rat hepatocytes from necrosis and apoptosis. Planta Med.

[21] Yoshioka K., Kataoka T., Hayashi T., Hasegawa M., Ishi Y., Hibasami H. (2000). Induction of apoptosis by gallic acid in human stomach cancer KATO III and colon adenocarcinoma COLO 205 cell lines. Oncol. Rep.

[22] Zheng G., Xu L., Wu P., Xie H., Jiang Y., Chen F., Wei X. (2009). Polyphenols from longan seeds and their radical-scavenging activity. Food Chem.

[23] Mota M.L., Thomas G., Barbosa Filho J.M. (1985). Anti-inflammatory actions of tannins isolated from the bark of *Anacardium occidentale* L. J. Ethnopharmacol.

[24] Uchiumi F., Maruta H., Inoue J., Yamamoto T., Tanuma S. (1996). Inhibitory effect of tannic acid on human immunodeficiency virus promoter activity induced by 12-*O*-tetra decanoylphorbol-13-acetate in Jurkat T-cells. Biochem. Biophys. Res. Commun.

[25] Feldman K.S., Sahasrabudhe K., Lawlor M.D., Wilson S.L., Lang C.H., Scheuchenzuber W.J. (2011). *In vitro* and *in vivo* inhibition of LPS-stimulated tumor necrosis factor-alpha secretion by the gallotannin beta-d-pentagalloylglucose. Bioorg. Med. Chem. Lett.

[26] Prakobwong S., Gupta S.C., Kim J.H., Sung B., Pinlaor P., Hiraku Y., Wongkham S., Sripa B., Pinlaor S., Aggarwal B.B. (2011). Curcumin suppresses proliferation and induces apoptosis in human biliary cancer cells through modulation of multiple cell signaling pathways. Carcinogenesis.

[27] Yeh R.D., Chen J.C., Yuanlai T., Yang J.S., Yu C.S., Chiang J.H., Lu C.C., Yang S.T., Yu C.C., Chang S.J. (2011). Gallic acid induces G0/G1 phase arrest and apoptosis in human leukemia HL-60 cells through inhibiting cyclin D and E, and activating mitochondria-dependent pathway. Anticancer Res.

[28] Nunez G., Benedict M.A., Hu Y., Inohara N. (1998). Caspases: The proteases of the apoptotic pathway. Oncogene.

[29] Brenner C., Kroemer G. (2000). Apoptosis. Mitochondria—The death signal integrators. Science.

[30] Susin S.A., Lorenzo H.K., Zamzami N., Marzo I., Snow B.E., Brothers G.M., Mangion J., Jacotot E., Costantini P., Loeffler M. (1999). Molecular characterization of mitochondrial apoptosis-inducing factor. Nature.

[31] Zanna C., Ghelli A., Porcelli A.M., Martinuzzi A., Carelli V., Rugolo M. (2005). Caspase-independent death of Leber’s hereditary optic neuropathy cybrids is driven by energetic failure and mediated by AIF and Endonuclease G. Apoptosis.

[32] Cregan S.P., Fortin A., MacLaurin J.G., Callaghan S.M., Cecconi F., Yu S.W., Dawson T.M., Dawson V.L., Park D.S., Kroemer G. (2002). Apoptosis-inducing factor is involved in the regulation of caspase-independent neuronal cell death. J. Cell Biol..

[33] Yu F.S., Yang J.S., Yu C.S., Lu C.C., Chiang J.H., Lin C.W., Chung J.G. (2011). Safrole induces apoptosis in human oral cancer HSC-3 cells. J. Dent. Res.

